# Extremes in Therapeutics

**Published:** 1866-08

**Authors:** 


### EXTREMES IN THERAPEUTICS.

Stahl, the founder of the *expectant* system of practice, was the first leader in *one-ideal* systems. Hahnemann rose next; but to make a new system—one differing from that which had already been instituted, and yet dealing kindly, gently and softly with the sick, and giving very little trouble to the physician—he found it necessary to promulgate a new Therapeutical theory. Infinitessimal doses struck the founder of this new system as the most potent quantity, and at the same time safe, economical and convenient.

Extreme views naturally lead to the adoption of one-ideal systems. A desire to discharge the duties of professional life with the least possible tax upon the time and exertions, leads to some system of expectant practice. On the other hand, a desire to do a great deal without the ability to discriminate between those cases requiring active medication and those that need nothing, or the disposition to exercise the necessary caution in the treatment of disease, is apt to lead to the opposite and still more injurious extreme. The fact is patent to the most careless and superficial observer, even outside the profession, that the constant drugging too often practiced, without any rational reason for such a course, tends rather to increase than palliate or cure disease. The comparison between expectant and over-drugging systems is generally prejudicial to the latter; hence, homœopathy is selected by the invalid as the less evil. "If he does me no good, he will do me no harm," consoles the Valetudinarian as he swallows the sweet morsel in the form of homœopathic pellets. Again, a propensity prevails, in and out of the profession, to make any important and useful medicinal agent, or course of treatment, applicable to every form of disease. Water, having been known to produce very decided and beneficial effects, is paraded by a certain sect as the only agent necessary in the treatment of all manner of diseases. So the discovery that the local application of pepper to certain slight and superficial inflammations of the mucous membrane of the throat, instead of increasing, actually allays the excitement, led to the ridiculous theory, promulgated by Thompson and Howard, that all manner of disease is best treated by stimulants—that "heat is life and cold is death." In accordance with this theory, inflammatory and febrile affections were considered subject to cure by, not only cerebral and nervous stimulants, but those acting most actively upon the heart itself.

Such one-ideal systems or wholesale theories are very convenient under certain circumstances. The charlatan who never knew, nor expects ever to become acquainted with the first principles of physiology and pathology, can very readily administer certain remedies under so simple and universal a theory—one which requires no discrimination, judgment, science or common sense in order to apply remedies in every case of disease, whether local or constitutional, specific or general, sthenic or asthenic. The learned and scientific physician also finds a convenience in being able to soothe his conscience with some such general theory, when—from an indisposition to perform the mental and physical labor necessary to investigate the pathological condition, nature and tendencies of the diseases presented, and apply such remedies as are suited to each individual case—he does not choose to take the usual laborious course of investigation and prescriptions. Such are prone to imagine a kind of unity of diseases, requiring for their cure, in the main, about the same course of treatment—that all are sthenic and necessarily require depletion or other antiphlogistic means; or, that asthenic tendencies always exist, and stimulants of every class are not only admissible, but highly beneficial always. This opinion of unity of disease, or identity of immediate cause, is not the production alone of the present age, but forms the characteristic peculiarities of writers who flourished more than a century ago—most of which, as in the present day, originated in misconception of the therapeutical action of remedies. In the first place, a great mistake is often made by practitioners in too readily concluding that the favorable termination of disease is necessarily ascribable to the effects of the remedies administered, when, in fact, patients often recover, not only without any assistance from the treatment instituted, but even in despite its injurious effects. Statistical tables, therefore, incautiously gotten up, often lead to the establishment of false theories and erroneous systems of practice. Many diseases have a regular period to run, and at the end of this time often terminate favorably without therapeutical assistance, while, in more violent cases, the only safety resides in the action of medicinal agents. Again, the medicinal properties of certain agents are not properly understood and appreciated. Stimulants, for instance, are often used as such, indiscriminately, without regard to the part of the body which they directly excite. A stimulant to the brain does not necessarily excite unduly the heart, and *vice versa.* The various stimulants of the nervous centres and antiphlogistic means are not, therefore, antagonistic, as is too often supposed. The fact, then, that alcohol, opium, etc., are admissible, and often useful in inflammatory diseases, is no evidence that depletion and other sedatives and refrigerants are not demanded. In pneumonia, for example, when from the violence of the disease the nervous energies are depressed, brandy and opium will restore the vital powers, while the usual antiphlogistic means are resorted to for the relief of the local phlegmasia.

